# Peptidylarginine Deiminase Inhibition Abolishes the Production of Large Extracellular Vesicles From *Giardia intestinalis*, Affecting Host-Pathogen Interactions by Hindering Adhesion to Host Cells

**DOI:** 10.3389/fcimb.2020.00417

**Published:** 2020-09-23

**Authors:** Bruno Gavinho, Bruna Sabatke, Veronica Feijoli, Izadora Volpato Rossi, Janaina Macedo da Silva, Ingrid Evans-Osses, Giuseppe Palmisano, Sigrun Lange, Marcel Ivan Ramirez

**Affiliations:** ^1^Programa de Pós-Graduação em Microbiologia, Parasitologia e Patologia, Departamento de Patologia, Universidade Federal do Paraná, Curitiba, Brazil; ^2^Programa de Pós-Graduação em Biologia Celular e Molecular, Departamento de Biologia Celular, Universidade Federal do Paraná, Curitiba, Brazil; ^3^Departamento de Parasitologia, Instituto de Ciências Biomédicas II, Universidade de São Paulo, São Paulo, Brazil; ^4^Departamento de Enfermagem, Centro Universitario Santa Cruz, Curitiba, Brazil; ^5^Tissue Architecture and Regeneration Research Group, School of Life Sciences, University of Westminster, London, United Kingdom; ^6^Departamento de Bioquímica e Biologia Molecular, Universidade Federal do Paraná, Curitiba, Brazil; ^7^Instituto Oswaldo Cruz, Rio de Janeiro, Brazil

**Keywords:** extracellular vesicles (EVs), large vesicle-like structures, small vesicles, PAD inhibitors, cannabiol, diarrhea, *Giardia intestinalis*, parasite host cell-interaction

## Abstract

*Giardia intestinalis* is a microaerophilic protozoan that is an important etiologic agent of diarrhea worldwide. There is evidence that under diverse conditions, the parasite is capable of shedding extracellular vesicles (EVs) which modulate the physiopathology of giardiasis. Here we describe new features of *G. intestinalis* EV production, revealing its capacity to shed two different enriched EV populations: large (LEV) and small extracellular vesicles (SEV) and identified relevant adhesion functions associated with the larger population. Proteomic analysis revealed differences in proteins relevant for virulence and host-pathogen interactions between the two EV subsets, such as cytoskeletal and anti-oxidative stress response proteins in LEVS. We assessed the effect of two recently identified inhibitors of EV release in mammalian cells, namely peptidylarginine deiminase (PAD) inhibitor and cannabidiol (CBD), on EV release from *Giardia*. The compounds were both able to effectively reduce EV shedding, the PAD-inhibitor specifically affecting the release of LEVs and reducing parasite attachment to host cells *in vitro*. Our results suggest that LEVs and SEVs have a different role in host-pathogen interaction, and that treatment with EV-inhibitors may be a novel treatment strategy for recurrent giardiasis.

## Introduction

*Giardia intestinalis* is a lumen dwelling pathogen in the vertebrate gut which is responsible for a worldwide waterborne diarrhea known as giardiasis. It affects millions of people worldwide and remains a neglected disease (Savioli et al., 2006; reviewed by Ankarklev et al., 2010; Cernikova et al., 2018). The life cycle of this flagellated parasite consists of two evolutionary stages: (i) the trophozoite, which adheres to the intestinal epithelium and multiplies by binary fission, and (ii) the infectious stage, the cyst, that is released through feces and is acquired by ingestion of food or water. *Giardia* has a simple cell biology, lacking organelles and a typical endosomal sorting complex required for transport, the ESCRT (Saha et al., 2018). The parasite accesses nutrients through specialized structures called peripheral vesicles (PV), and the secretion of cyst wall proteins is operated by the encystation-specific vesicles (ESVs) which are absent in non-encysting trophozoites (Gottig et al., 2006). The virulence factors involved in Giardia pathogenesis have been described, including the cysteine proteases, which have been well-studied. These are involved in intestinal epithelial junctional complex disruption and degradation of host immune factors, e.g., cytokines (Bartelt and Sartor, 2015; Cotton et al., 2015; Liu et al., 2018). The parasite is also capable of evading the adaptive immune defense through antigenic variation (Prucca et al., 2008; Serradell et al., 2016). Yet, pathogenesis remains to be fully understood. Finally, chronic infection is a significant concern in giardiasis, as it represents nutritional implications for children and immunocompromised individuals (Halliez and Buret, 2013; Bartelt and Sartor, 2015).

Adaptions by protozoans for survival in the host involve sophisticated forms of host-pathogen communication (Cipriano and Hajduk, 2018). Importantly, many reports have described the release of extracellular vesicles (EVs) from pathogens to be relevant to the disease status (Cwiklinski et al., 2015; Coakley et al., 2017). EVs are found in most biological fluids and are 30–1,000 nm lipid-bilayer vesicles, which are shed from cells and transport a range of biomolecules, including protein and genetic material, participating in cell communication in physiological and pathophysiological processes (Coakley et al., 2015; Nawaz et al., 2016; Maas et al., 2017; Ramirez et al., 2018; Ryu et al., 2018; van Niel et al., 2018).

Our group has previously described EV release of *G. intestinalis* and established that giardial EVs are involved in host-pathogen interactions via immunomodulation and trophozoite persistence (Evans-Osses et al., 2017). In addition, Ma'ayeh et al. (2017) recovered EVs from the secretome of an axenic culture. In recent years, there has been a growing interest in improving the current knowledge of the nature, constitution, and biogenesis of all secreted EVs. In the last few decades, the term “exosomes” was proposed to refer to smaller EVs (sEVs) of multivesicular bodies origin (<100 nm diameter). Concomitantly, larger EVs (LEVs) called “microvesicles” (~100–1,000 nm) are released directly from the plasma membrane (Tkach et al., 2018; Mathieu et al., 2019). Lately, the field of EVs has debated the need to accurately separate EV subtypes to investigate functional relevance (Tkach et al., 2018). There are current limitations in techniques that analyze EVs and possibly, a relation of size and biological function is an appropriate step toward characterization (Moyano et al., 2019). Enrichment of subpopulations of EVs from samples is acquired based on differential centrifugation steps: large EVs (LEVs) are obtained at speeds lower than 20,000 × g and small EVs (SEVs) are pelleted at 100,000 × g in a further ultracentrifugation step. Formerly, microvesicles and exosomes, particularly present in LEV and SEV, respectively, are considered the main EV subpopulations. Exosomes are the smallest population, continually produced in the late endosome and liberated through the fusion of multivesicular bodies within the plasma membrane. Microvesicles are particles of a larger size produced though budding from the plasma membrane under stress mediated by scramblase, calpain, and Ca^2+^ liberation (Morrison et al., 2016; Nawaz and Fatima, 2017). Different roles for these EV subpopulations remain a focus of ongoing investigations; are all EVs phenotypically relevant and/or similar?

While it is known that EVs are released by multiple mechanisms, some advances in the understanding of their biogenesis has been elucidated via studies on the peptidylarginine deiminase (PAD)-mediated pathway of EV release in diverse taxa (Kholia et al., 2015; Kosgodage et al., 2017, 2018a, 2019a; Lange et al., 2017). PADs are phylogenetically conserved enzymes from bacteria to mammals (Vossenaar et al., 2003; Magnadottir et al., 2018; Kosgodage et al., 2019a), including in *Giardia* (arginine deiminase GiADI; Trejo-Soto et al., 2016). PADs catalyze post-translational deimination by irreversibly changing arginine into citrulline in a calcium-catalyzed manner in target proteins, affecting their folding and function (Vossenaar et al., 2003; György et al., 2006). PADs are involved in pathophysiological processes and their up-regulation and associated increase in deiminated proteins is associated with various pathologies including autoimmune and neurodegenerative diseases, as well as cancer (Vossenaar et al., 2003; Wang and Wang, 2013; Witalison et al., 2015; Lange et al., 2017; Kosgodage et al., 2018a; Uysal-Onganer et al., 2020). While exact roles for PADs in EV biogenesis and release remain to be fully elucidated, effects on cytoskeletal, nuclear, and mitochondrial proteins have been reported (Kholia et al., 2015; Kosgodage et al., 2018a; Uysal-Onganer et al., 2020). As pharmacological PAD-inhibitors have previously been shown to be potent inhibitors of EV release in various cancer cells and in bacteria, and modulators of EV cargo (Kholia et al., 2015; Kosgodage et al., 2017, 2018a, 2019a), we sought to investigate a phylogenetically conserved influence of PAD-inhibitors on the EV production of our parasite model.

In addition, cannabidiol (CBD), a phytocannabinoid derived from *Cannabis sativa* (Mechoulam et al., 2002), was recently identified as a potent EV-inhibitor in cancer cells as well as in bacteria (Kosgodage et al., 2018b, 2019b,c), indicating a phylogenetically conserved function in EV regulation. As cannabinoids have previously been associated with anti-parasitic functions (Nok et al., 1994; Croxford et al., 2005; Roulette et al., 2016) and the reduction of bacterial antibiotic resistance via the inhibition of bacterial EVs/MVs (Kosgodage et al., 2019c), we sought to identify whether EV release from *Giardia* may be affected by CBD, thus elucidating a novel aspect of CBD function on *Giardia*-host interaction.

Here, we report that *G. intestinalis* produces two populations of EVs that differ in size. The larger EV population had a significant effect on parasite-host adhesion *in vitro* and was significantly reduced by both PAD-inhibitor and CBD. In addition, treatment with PAD-inhibitor selectively prevented parasite LEV production.

## Methods

### *G. intestinalis* Isolates and Cell Culture

*G. intestinalis* isolate WB (ATCC 50803) were grown in TYI-S-33 medium (Keister, 1983) supplemented with 10% heat-inactivated adult bovine serum with 1% Penicillin/Streptomycin 1000 U (Gibco™) and 0.5 mg/ml bovine bile (ThermoFisher™) at 37°C under microaerophilic conditions. The cultures were maintained in polystyrene tubes (BD Biosciences™) (13 mL) until confluence (1 × 10^6^ cells mL). and thereafter sub-cultured, each for 72 h. Human colorectal adenocarcinoma cells, Caco-2 cells (ATCC CRL-2102) were cultured in RPMI-1640 supplemented with 10% fetal bovine serum, 2 mM r-glutamine, and 1% Penicillin/Streptomycin 1000 U (Gibco™). Cells were incubated at 37°C in 5% CO_2_ until a confluent cell monolayer was reached.

### EV Isolation

For parasite EV isolation, parasites from confluent culture tubes were detached by chilling for 15 min on ice, centrifuged twice (600 × g/5 min) and the pellets suspended with fresh TYI-S-33 without adult bovine serum (ABS). Parasites were then counted using a hemocytometer, and diluted to 1 × 10^6^ per sample according to Evans-Osses et al. (2017). Samples were distributed to 1.5 mL microtubes (final volume of 1 mL) and 1 mM of CaCl_2_ was added for EV induction and the tubes were then incubated for 1 h at 37°C. Then, EV pellets were collected via step-wise centrifugation: first, at 600 × g/5 min; 4,000 × g/30 min to eliminate cellular debris, thereafter the supernatant was centrifuged at 15,000 × g for 1 h and the resulting pellet (LEV) was resuspended in phosphate buffered saline (PBS). The remaining supernatant was then ultracentrifuged for 100,000 × g for 4 h, and the resulting EV-containing pellets (SEV) were resuspended in PBS (1x). Both samples were kept at 4°C until further use.

For mammalian EV purification, Caco-2 cells were cultured in T25 flasks until confluence, and then the medium was removed. Cultures were washed twice with fresh RPMI-1640 and kept for 1 h with medium omitting ABS, to avoid contamination of EVs from ABS. The supernatant was processed in the same manner as described for the parasite. EVs were stored at 4°C until further use for host-pathogen interaction assays.

### EV Quantification and Characterization

EVs were in the first instance quantified based on their protein concentrations using the Micro BCA assay (ThermoFisher™). For Nanoparticle tracking analysis (NTA, Nanosight, Malvern, U.K.), each sample was diluted 1:100 in PBS (1x) and subjected to a NS300 Nanosight (Malvern™, U.K.), with readings performed in triplicate during 60 s videos at 10 frames per second at room temperature, with the following parameters: camera shutter −1492, camera gain −512, detection threshold −10. The resulting replicate histograms were averaged for presentation in box-plots.

### Treatment of Trophozoites With EV-Inhibitors

Inoculum of 10^6^ trophozoites per group (triplicates) were stimulated with 1 mM CaCl_2_ in 1.5 mL microtubes for the production of EVs, with or without EV-inhibitors. Experimental groups were as follows: medium only (control), 100 or 50 μM PAD-inhibitor Cl-amidine (a kind gift from Prof Paul Thompson, UMASS), or with 5 or 10 μM CBD (90899_SIAL, Sigma-Aldrich™). After 60 min of incubation (37°C), supernatants were processed following the protocol described above for EV isolation.

### Trophozoites Growth Curves

A growth curve was generated from the parasites submitted to vesiculation at different time intervals (1, 3, or 6 h).

Inoculums of 100 μL at 1 × 10^5^ trophozoite/mL from each group were resuspended in a complete TYI-S-33 medium, added to polystyrene culture tubes, and maintained at 37°C, for an overall time period of 96 h. Every 24 h, the tubes were chilled on ice for 15 min to promote trophozoite detachment, and aliquots of 10 μL were subsequently taken from the culture tubes to count *Giardia* trophozoites on a hemocytometer under a Bioval optical microscope.

Following the protocol described above, we also maintained growth curves from trophozoites treated with Cl-amidine (100 μM), Cannabidiol (CBD) (10 μM), and WT (control).

### Host-Pathogen Interaction Assay Exposed to EV-Inhibitors

Caco-2 cells were seeded in 24-well plates and grown to 100% confluence. Inoculations of 5 × 10^5^ trophozoites per group were transferred to the cell monolayer for 3 h (37°C) in a final volume of 1 mL/well. The following groups were investigated: medium only (control), 10 μM CBD, and 100 μM Cl-amidine. After incubation, trophozoite quantification was performed by centrifuging non-adherent parasites from the supernatant and counting them from the pellet using a hematocytometer. The percentage of trophozoites adhering to Caco-2 cells was subsequently calculated according to Cotton et al. (2014).

Experiments were also conducted with the two different EV populations. In those cases, the co-cultures were treated with 7 or 14 μg LEVs and 7 or 14 μg SEVs. In order to understand whether the EV-modulatory effect of Cl amidine or CBD was affected by a Ca, Mg, or IP3 lysozome pathway, we performed an independent experiment where we treated trophozoites with chelating agents (5 mM EDTA or EGTA), and an inhibitor of the PI3K signaling pathway, wortmanin (100 nM). We have tried to obtain preliminary findings to understand whether there might be a synergism of these agents with either the Cl-amidine or CBD inhibitor. As Cl-amidine was the more effective EV inhibitor, we assessed the compounds only in the presence of 100 μM Cl-amidine. In a separate experiment, EVs were subjected to heat inactivation (65°C/30 min), as described in Salomon et al. (2014), and then used as negative controls of EVs to treat trophozoites with or without exposure to Cl-amidine (100 μM).

In independent experiments to assess the effects of mammalian (Caco-2 derived) EVs on trophozoite adhesion, the same host-pathogen interaction assay was used. Experimental groups were as follows: 7 or 14 μg Caco-2 cell SEVs, 7 or 14 μg Caco-2 cell LEVs, and the negative control with medium alone.

### Influence of Protease Inhibitor on Host-Pathogen Interaction

The influence of a protease inhibitor was assessed for parasite adhesion to Caco-2 cells. For this, 5 × 10^5^ trophozoites were seeded into Caco-2 cell monolayers at 37°C. The co-culture was treated with 1 mM iodoacetamide (IAA, cysteine protease inhibitor), with or without 3.5 μg LEVs (3 h), and the negative control with medium alone. Adhesion estimation was performed as in the host-pathogen interaction assay.

### Sample Preparation for Mass-Spectrometry Based Proteomics

Ev preparations were resuspended in 8M Urea containing a protease inhibitor cocktail (Sigma-Aldrich). Samples were subjected to six cycles of freeze and thaw before being quantified by a fluorometric assay using the Qubit™ Protein Assay Kit (Invitrogen, Carlsbad, CA) platform according to the manufacturer's instructions. A total of ± 100 μg proteins were reduced with 10 mM Dithiothreitol (DTT) at 30°C for 45 min, followed by alkylation of cysteine residues with 40 mM of iodoacetamide (IAA) for 30 min at room temperature in the dark. Thereafter, the samples were incubated with DTT 5 mM for 15 min at 30°C. The samples were digested with trypsin (Promega, Cat#: V5111) (1:50, Enzyme: Substrate) for 18 h at 30°C. Following digestion, all reactions were acidified with 10% (v/v) trifluoroacetic acid (1% v/v final concentration) to stop proteolysis. The samples were centrifuged for 10 min at 12,000 × g to remove insoluble materials. The tryptic peptides were desalted prior to LC-MS analysis using two C18 disks (3 M Empore TM C18 extraction disk) stage-tips.

### Nanoflow Liquid Chromatography Coupled to Mass-Spectrometry (nLC-MS/MS) Analysis

The nLC-MS/MS analysis was performed using an Easy Nano LC1000 (Thermo) HPLC coupled with an LTQ Orbitrap Velos (Thermo), where 10 μL of the sample was applied using a 300 nL/min flow rate of Mobile phase A (5% ACN 0.1% formic acid) in a C18 EASY-column (2 cm × 5 μm × 100 μm; 120 Å pore, Thermo) and separated in a C18 PicoFrit PepMap (10 cm × 10 μm × 75 μm; 135 Å pore, New Objective), over 105 min using a linear gradient 2–30% of mobile phase B (100% ACN; 0,1% formic acid). The eluted peptides were ionized using electrospray. The top 20 most intense precursor-ions with charge-state = 2 were fragmented using CID at 35 normalized collision energy and 10 ms activation time. The MS scan range was set between 350 and 1,500 m/z, the MS scan resolution was 60.000, the MS1 ion count was 1 × 10e6, and the MS2 ion count was 3 × 10^4^. The experiments were analyzed in biological triplicates.

### Protein Identification

Raw files were imported to MaxQuant version 1.5.3.8 with the Andromeda search machine and searched against the Uniprot *Giardia intestinalis* strain ATCC 50803 WB clone C6 database (November 28, 2019 release; 7,156 entries) with 20 ppm for MS/MS. The followed parameters were used: carbamidomethylation of cysteine (57.021464 Da) as a fixed modification, and oxidation of methionine (15.994915 Da) and N-terminal acetylation protein (42.010565 Da) were selected as variable modifications. Enzyme specificity was set to full trypsin with a maximum of two missed cleavages. The minimum peptide length was set to 7 amino acids.

### EV Staining

For uptake assays, EVs were stained and tested with carboxyfluorescein succinimidyl ester (CFSE, ThermoFisher^TM^), or with the lipophilic dye PKH-26 (SigmaAldrich™). For CFSE labeling, 1 μL of the fluorescent dye was diluted with both EV populations in 1 mL PBS. For PKH-26, 2 μL of the fluorescent dye were diluted in 1 mL of diluent C and both EV populations were diluted 1/40 in diluent C. Both dilutions were mixed together at a volume ratio of 1:1. For both fluorescent dyes, labeling was continued for 15 min at room temperature in the dark. The reaction was stopped by adding 1 mL Fetum bovine serum, and samples were then washed in PBS (1x), and ultracentrifuged at 15,000 × g for 1 h to obtain LEVs and at 100,000 × g for 4 h for the collection of SEV, as before.

### Parasite EVs Uptake by Caco-2 Cells

Caco-2 cells were incubated on sterile coverslips at 37°C in 5% CO_2_ with 7 or 14 μg of PKH26-labeled giardial EVs (ThermoFisher™) for 1 h. Caco-2 cell monolayers were also labeled for nuclei (DAPI, blue—ThermoFisher™). After incubation, the cells were washed three times in cold PBS (1x), and fixed with 4% paraformaldehyde. Coverslips were washed with PBS (1x) and mounted with 10 μl of a 50% glycerol solution. Internalized EVs were detected by confocal microscopy (Nikon A1R HD Multifoton Confocal). Images were processed by Image J software (v. 1.48—open source, Schneider et al., 2012). Fluorescence intensity of two images per sample was obtained in a duplicate experiment, and corrected cellular fluorescence was estimated as in McCloy et al. (2014).

### Cytotoxicity Assay of EV-Inhibitors Toward Caco-2 Cells

Caco-2 cells were seeded into a 96-well plate and grown at 37°C in 5% CO_2_ until confluence in RPMI-1640 medium containing 10% fetal bovine serum and 1% penicillin/streptomycin (10 000 UI). Cells were treated with 10 μM albendazole (ABZ, positive control), 100 μM Cl-amidine or 10 μM CBD, in a final volume of 100 μL per well. After 48 h, wells were washed with 100 μL of PBS (1x). Cells were fixed with absolute methanol (50 μL) for 10 min, after which 50 μL of crystal violet 0.2% in ethanol/water (2% V/V) were added to each tube. After 2 min, the wells were exhaustively washed with 200 μL of PBS (1x). Elution was made with a sodium citrate solution (0.05 μmol, 10 min), and absorbance was determined at 540 nm on a plate spectrophotometer, as described in Missina et al. (2018).

### Statistical Analysis

Statistical analysis of data was performed with GraphPad Prism 6 Software using one or two-way ANOVA test. Values are represented as means ± standard errors of the means (SEM), acquired in biological triplicates. The normality of the data was assessed prior to analysis. *P* = 0.05 were defined as significant.

## Results

### *Giardia* EV Biogenesis: Identification of Two Distinct EV Populations

**Figure 1:**
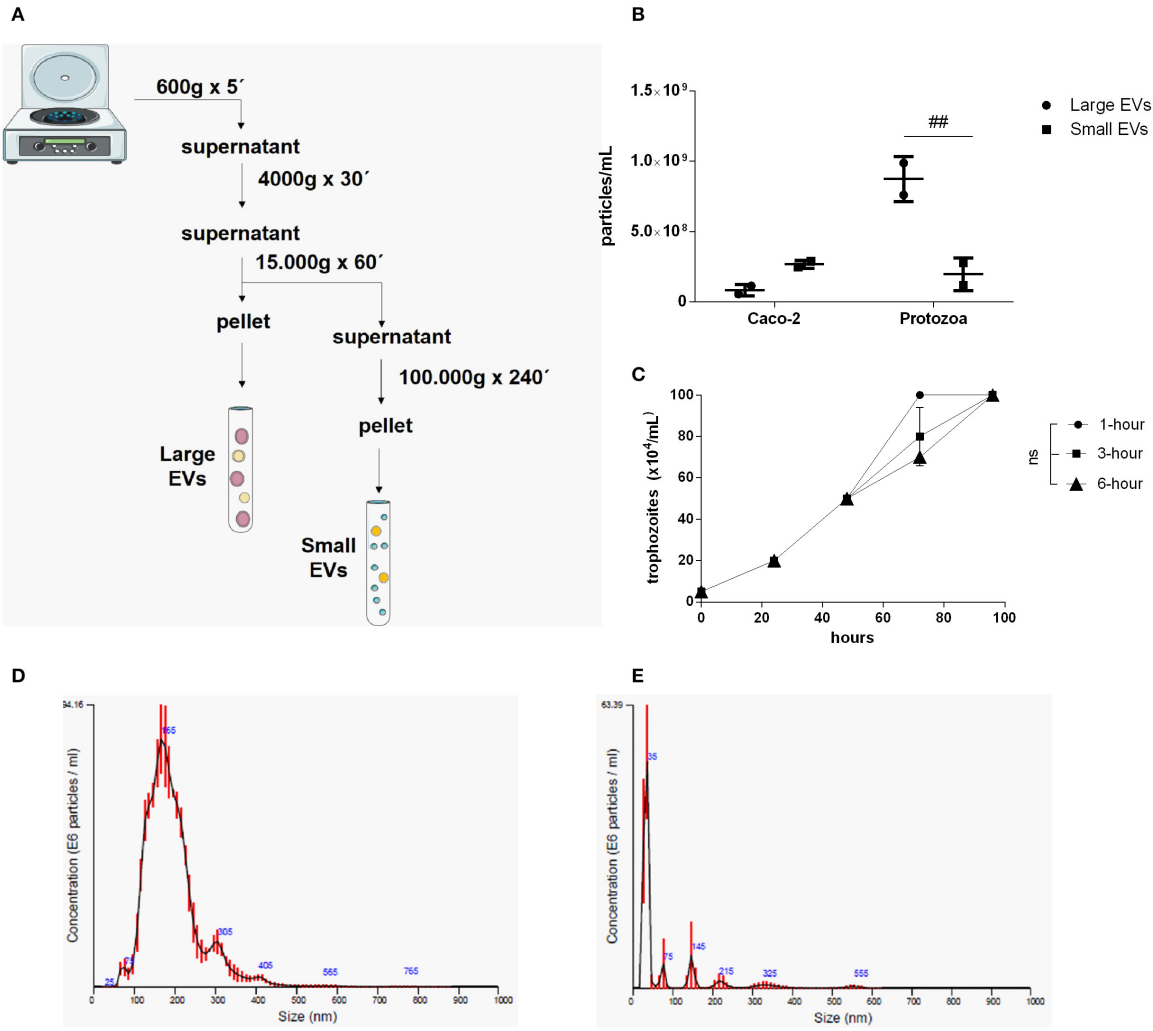
Isolation and characterization of two distinct EV populations produced from *Giardia intestinalis*. **(A)** The protocol for the isolation of LEVs and SEVs based on differential centrifugation. **(B)** Quantification of vesicle numbers from nanoparticle tracking analysis (NTA). **(C)** Time-course for culture confluence of trophozoites induced to produce EVs for 1, 3, and 6 h, respectively. **(D,E)** Particle size estimated by NTA for LEVs **(D)** and SEVs **(E)**. Data are representative of at least three independent experiments and represented as means ± SEM. ^*##*^*P* = 0.01, vs. the corresponding group, indicated by line; ns, not significant.

Two different EV sub-populations were isolated from *G. intestinalis*. The previous protocol (Evans-Osses et al., 2017) was slightly modified (addition of a 15,000 × g step for 1 h, increased ultracentrifugation time to 4 h) to separate putative large extracellular (LEVs) and small extracellular vesicles (SEVs) from the total EVs described before (Figure 1A). Using this method, LEVs were recovered at 15,000 × g and SEVs at 100,000 × g. Trophozoites were incubated with calcium chloride, an enhancer of EV production. This protocol was performed for parasite and mammalian host cells (Caco-2). Nanoparticle tracking analysis (NTA; Figure 1B) showed a higher yield of parasite EVs compared with Caco-2 cells (~3-fold higher), as also confirmed by protein detection (Supplementary Figure 1). In addition, *Giardia* was found to be capable of shedding more LEVs (~3-fold higher) compared with SEVs. LEV and SEV fractions showed a mean vesicle diameter of 187.6 and 67.7 nm, respectively (Figures 1D,E). Growth assessment was performed after stimulating trophozoites to produce EVs for 1, 3, and 6 h, respectively, following the growth curve in a complete medium by 96 h at 37°C in 5% CO_2_ (Figure 1C). There were no significant differences observed on the growth-curve from the different groups.

### PAD-Inhibitor and CBD Treatment Affects *Giardia* EV Biogenesis

**Figure 2:**
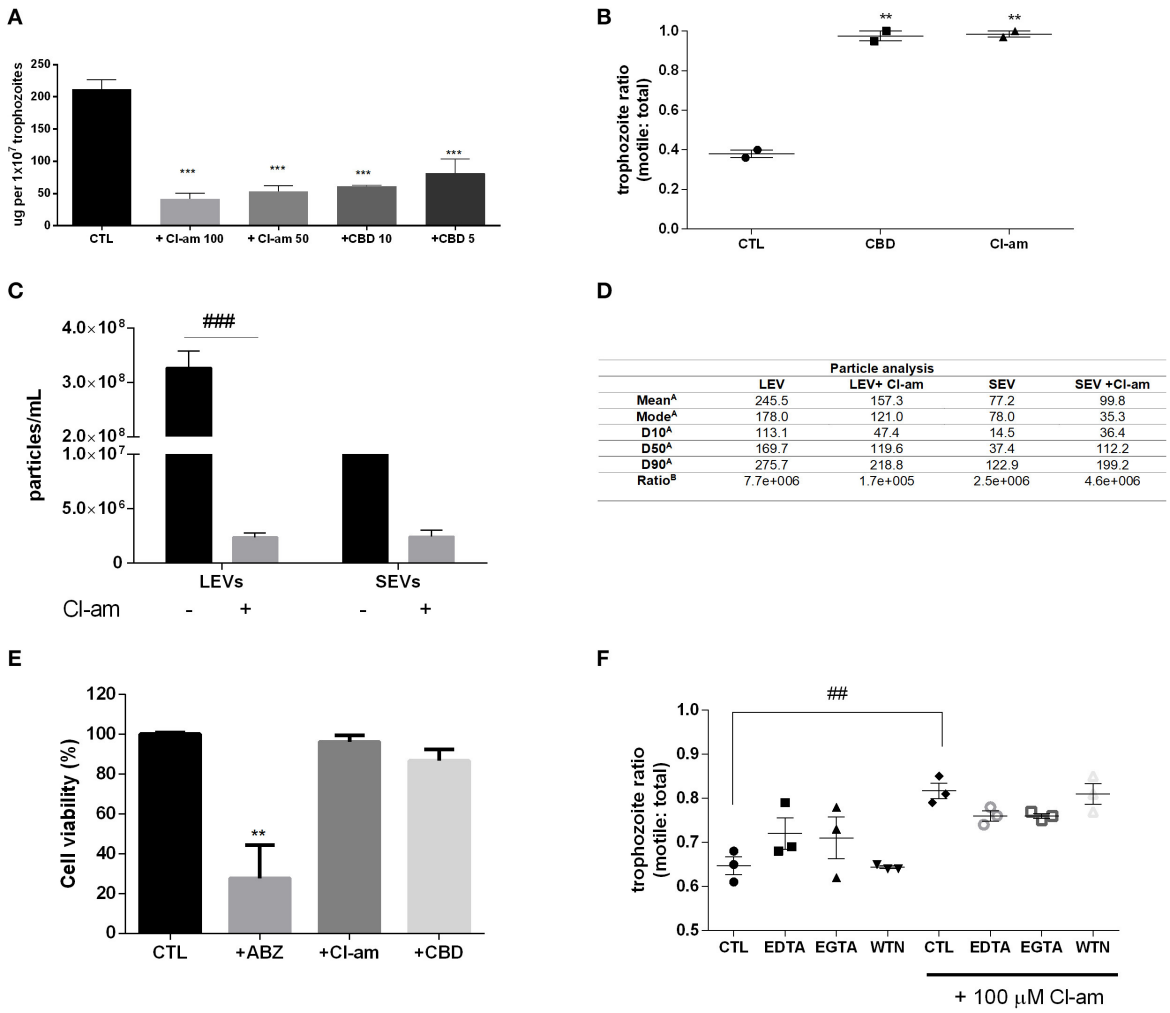
EV-inhibitors Cl-amidine and CBD decrease the production of LEVs secreted from *G. intestinalis*. **(A)** EV protein quantification post-treatment with Cl-amidine (Cl-am; 100 and 50 μM) or CBD (10 and 5 μM). **(B)** Host-pathogen assay concomitant to treatment with PAD-inhibitor (100 μM) or CBD (10 μM). **(C)** *Giardia* EV concentration estimation by nanoparticle tracking analysis (NTA) post-treatment with 100 μM Cl-am. **(D)** Particle analysis of the parasite distinct EV populations [**(A)**, EV size estimation of the distinct EV populations by NTA (nm); **(B)**, yield particle/μg of protein obtained in 1 × 10^6^ cells]. **(E)** Cytotoxic effects of the EV-inhibitors on Caco-2 cell monolayers. Cells were incubated for 48 h with ABZ, Cl-am, and CBD at 10, 100, and 10 μM, respectively, or with culture medium. Cell viability was determined by the crystal violet method. **(F)** Effect of Cl-amidine in host-pathogen assay after treatment with EDTA (5 mM), EGTA (5 mM), and wortmannin (WTN; 100 nM). Data are representative of at least three independent experiments and represented as means ± SEM. **P = 0.01, ***P = 0.001 compared to control group (CTL). ^*##*^*P* = 0.01, ^*###*^*P* = 0.001; vs. the corresponding group, indicated by line.

*G. intestinalis* trophozoites were treated with the PAD-inhibitor Cl-amidine or CBD, respectively, to assess the effects on EV release. Both compounds were able to significantly reduce production of EVs from *Giardia* (Figure 2A). In addition, we assessed the ability to block host-pathogen interactions following treatment with the EV-inhibitors. Indeed, both compounds were capable of decreasing trophozoite adhesion to the Caco-2 cell monolayer (Figure 2B).

We further assessed if Cl-amidine affected the release of SEVs and LEVs equally. Concentration of LEVs by NTA estimated a significant difference between treated compared to non-treated groups (~100-fold higher), while there was no difference observed for SEVs between treated vs. control non-treated parasites (Figure 2C). The mean diameter (nm) of EVs released in the presence of Cl-amidine was significantly reduced for LEVs as follows: LEV (245.5), + Cl-am LEV (157.3), SEV (77.2), +Cl-am SEV (99.8). Values of vesicles from each population, as well as ratio particle/ug protein are shown in Figure 2D. EVs were exposed to heat inactivation before treatment of co-cultures; trophozoites treated with Cl-am and EVs showed a higher attachment to Caco-2 cells when compared to parasites treated with Cl-am and heat-inactivated EVs (Supplementary Figure 2).

We performed a toxicity test using mammalian cells, and PAD-inhibitor or CBD had no cytotoxic effects at 48 h post-treatment, compared with albenzadole, one of the common drugs used for giardiasis treatment (Figure 2E). Trophozoites exposed to the EV-inhibitors for 1 h in the vesiculation protocol were resuspended in culture tubes and did not show any significant difference in growth-curves, compared with the control culture (Supplementary Figure 3). We furthermore treated trophozoites with chelating agents (EDTA,a Ca^2+^/Mg^2+^ chelator, EGTA,Ca^2+^ chelator), and WTN (a PI3K inhibitor) in a host-pathogen assay. These compounds did not affect parasite adhesion (Figure 2F).

### LEVs Derived From Parasite, but Not Host EVs, Restore the Lack of Adhesion to Host Cells for *G. intestinalis* Trophozoites, Treated With EV-Inhibitors

**Figure 3:**
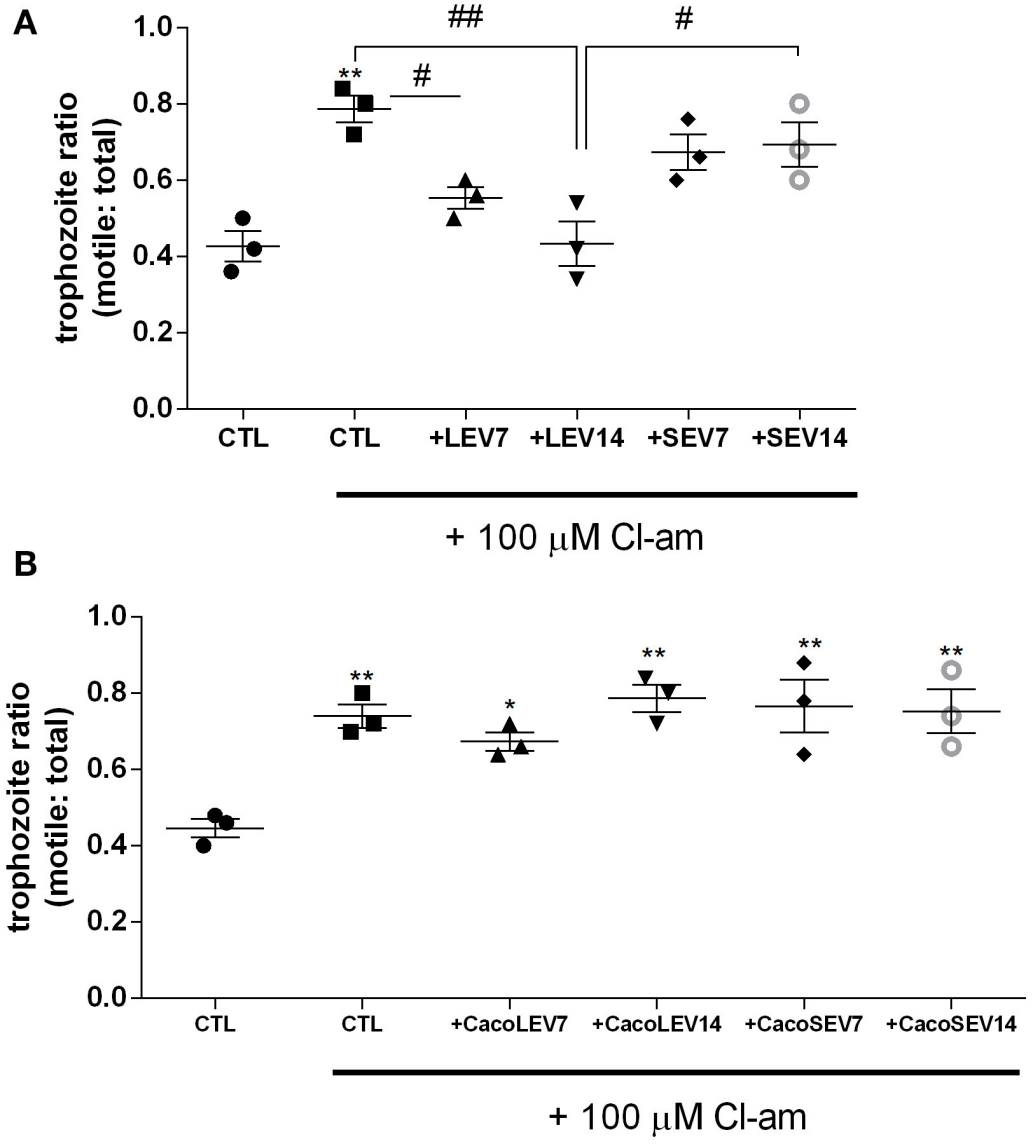
Parasite derived EVs are selectively involved with *Giardia* adhesion to host cells. **(A)** Adhesion assay following treatment with distinct parasite EV populations and 100 μM Cl-amidine. **(B)** Host-pathogen assay after treatment of Caco-2 cell monolayer with Cl-amidine and incubation with mammalian cell derived EVs. Data are representative of at least three independent experiments and represented as means ± SEM. *P = 0.05, **P = 0.01; compared to control group (CTL). ^#^*P* = 0.05, ^*##*^*P* = 0.01; vs. the corresponding group, indicated by line.

Due to the identification of the two EV populations, LEVs and SEVs, in *G. intestinalis*, we sought to verify whether these EV subpopulations had the same phenotype effect on host cell adhesion. For this assay, two concentrations (7 or 14 μg) from both EV populations, corresponding to the EV release for this amount of parasite in *in vitro* vesiculation, were used. LEVs derived from the parasite were capable of restoring the adherence phenotype following treatment with Cl-amidine, in a dose-dependent manner (Figure 3A). In contrast, no effect was observed in the SEVs treated groups. These results suggest that physical properties related to adherence can be found in the larger *Giardia* EVs and therefore, EVs produced by the parasite may selectively influence its phenotype.

We next assessed if the EVs from the host contribute to the adherence process to host cells. Confluent Caco-2 cell monolayers were washed and thereafter treated with Cl-amidine, and trophozoites were added to the wells, followed by treatment with Caco-2 cell-derived EVs (Figure 3B). Opposed to what was observed for the trophozoite EVs, neither mammalian LEVs nor SEVs had a phenotypical effect on trophozoite adhesion.

Moreover, we sought to investigate the influence of cysteine peptidase inhibitors on parasite adhesion to Caco-2 cells, treated with IAA. The cysteine protease inhibitor caused a significant reduction in trophozoite adhesion to host cells (Supplementary Figure 4) but this effect was reduced when the parasites were incubated with LEVs from the parasites, indicating that protease activity is not related to the effect on adhesion, observed for EVs.

### EVs Subtypes Contain Different Protein Profiles

Proteomic analysis of SEVs and LEVs revealed some differences in protein profiles (Supplementary Table 1). A total of 138 quantified proteins were identified in the LEV and SEV groups. Seventy-seven proteins were identified only in the LEV group and 19 proteins were exclusively found in the SEVs. Important products related to the *Giardia* genre were found common to both EVs, including antigenic Variable Surface Proteins (VSPs), giardins, cathepsin B, and other virulence factors (arginine deiminase, ornithine carbamoyltransferase).

**Figure 4:**
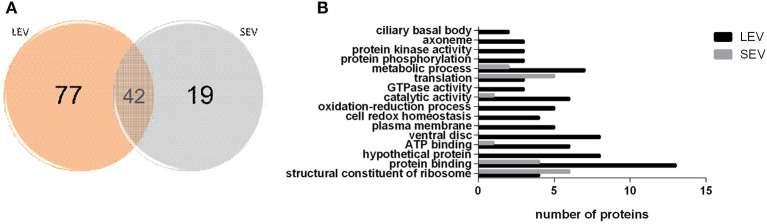
EV subtypes derived from *G. intestinalis* differ in protein cargo. **(A)** Venn diagram of peptides identified in LEV and SEV. (**B)** Gene ontology for proteins identified in LEV and SEV.

**Figure 5:**
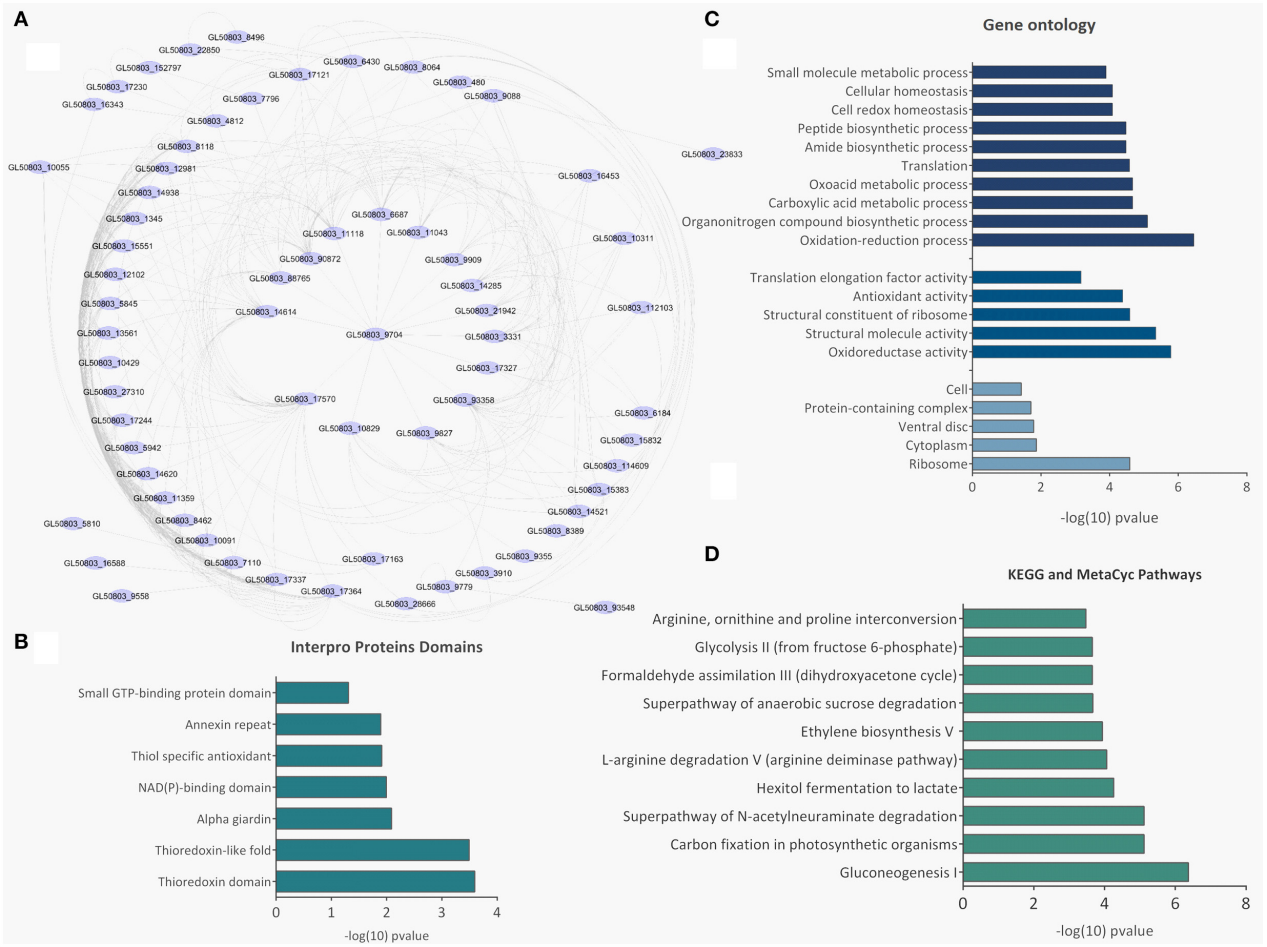
Bioinformatic analysis of SEV and LEV proteins. **(A)** Protein-protein interaction networks. **(B)** Gene ontology. **(C)** Enriched metabolic pathways. **(D)** Pfam domains identified in the total SEV and LEV protein datasets.

**Figure 6:**
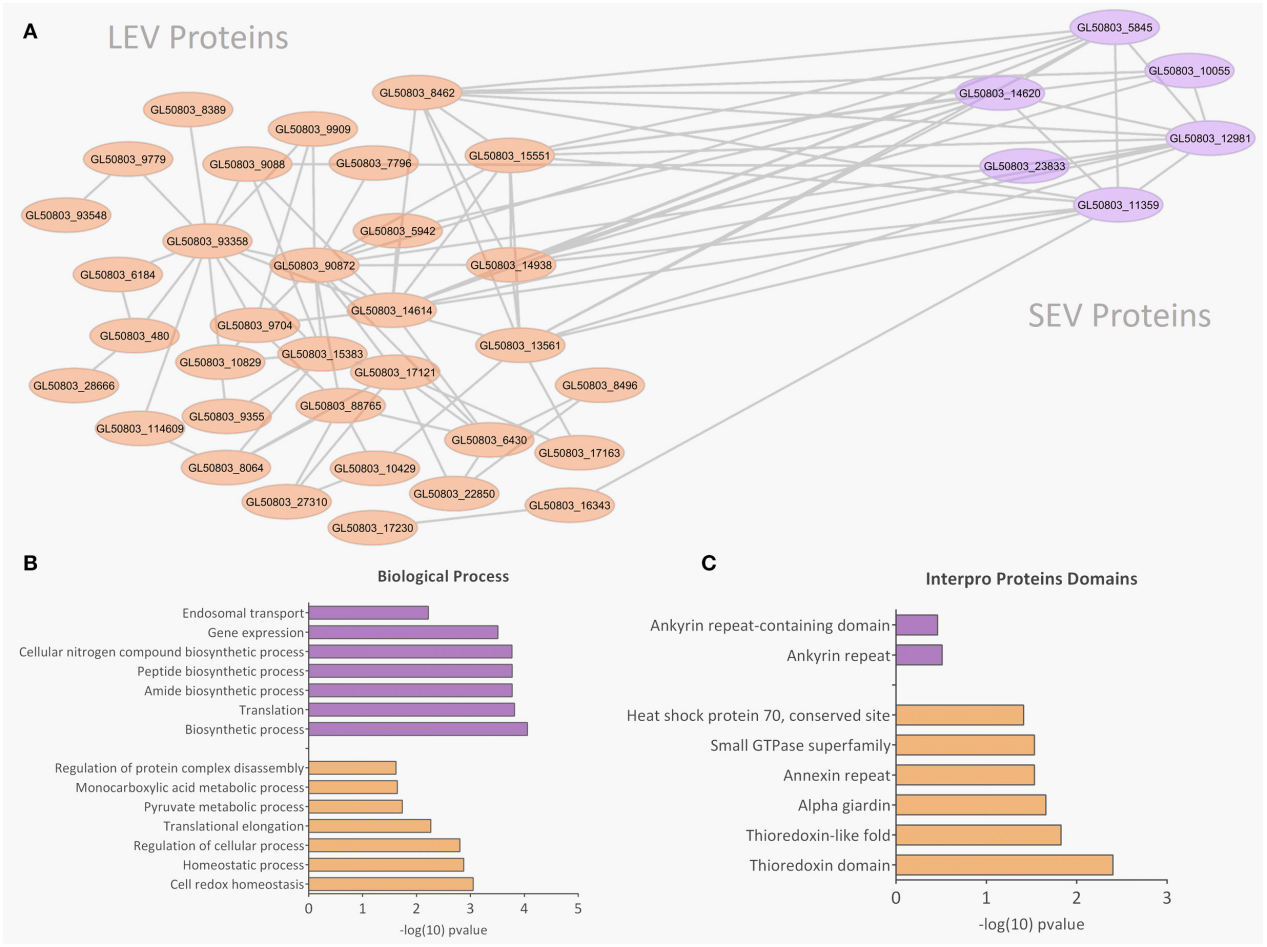
Protein-protein interaction, biological processes, and Pfam domains related to SEV and LEV exclusive proteins. Orange and purple color indicates exclusive proteins identified in LEV and SEV, respectively. **(A)** Protein-protein interactions between the exclusive proteins of LEV and SEV. **(B,C)** Biological processes and Pfam enriched domains are shown, respectively.

There were 77 proteins exclusively found in LEVs related to cytoskeleton composition and protein binding (Figures 4A,B). Products identified in LEV are associated with the cytoskeleton, as well as oxidative stress responses, such as Peroxiredoxin-1 (Supplementary Table 2). SEVs exclusively contained 19 proteins of which some related to ribosome metabolism. Bioinformatic analysis of the *Giardia* EVs proteome suggests the enrichment of enzymatic and cytoskeletal products, metabolic processes, as well as stress response to oxygen (Figure 5). Analysis of LEV and SEV specific proteins revealed a distinct cellular sub-localization, such as cytoplasm, nucleus, and plasma membrane, while SEVs proteins were mostly cytoplasmic. Protein-protein interaction and gene ontology analysis of LEV and SEV unique proteins revealed different biological processes and different structural domains (Figures 6A–C).

### Host-Pathogen Interactions: Both SEV and LEV *Giardia* Types Are Internalized by Mammalian Cells

**Figure 7:**
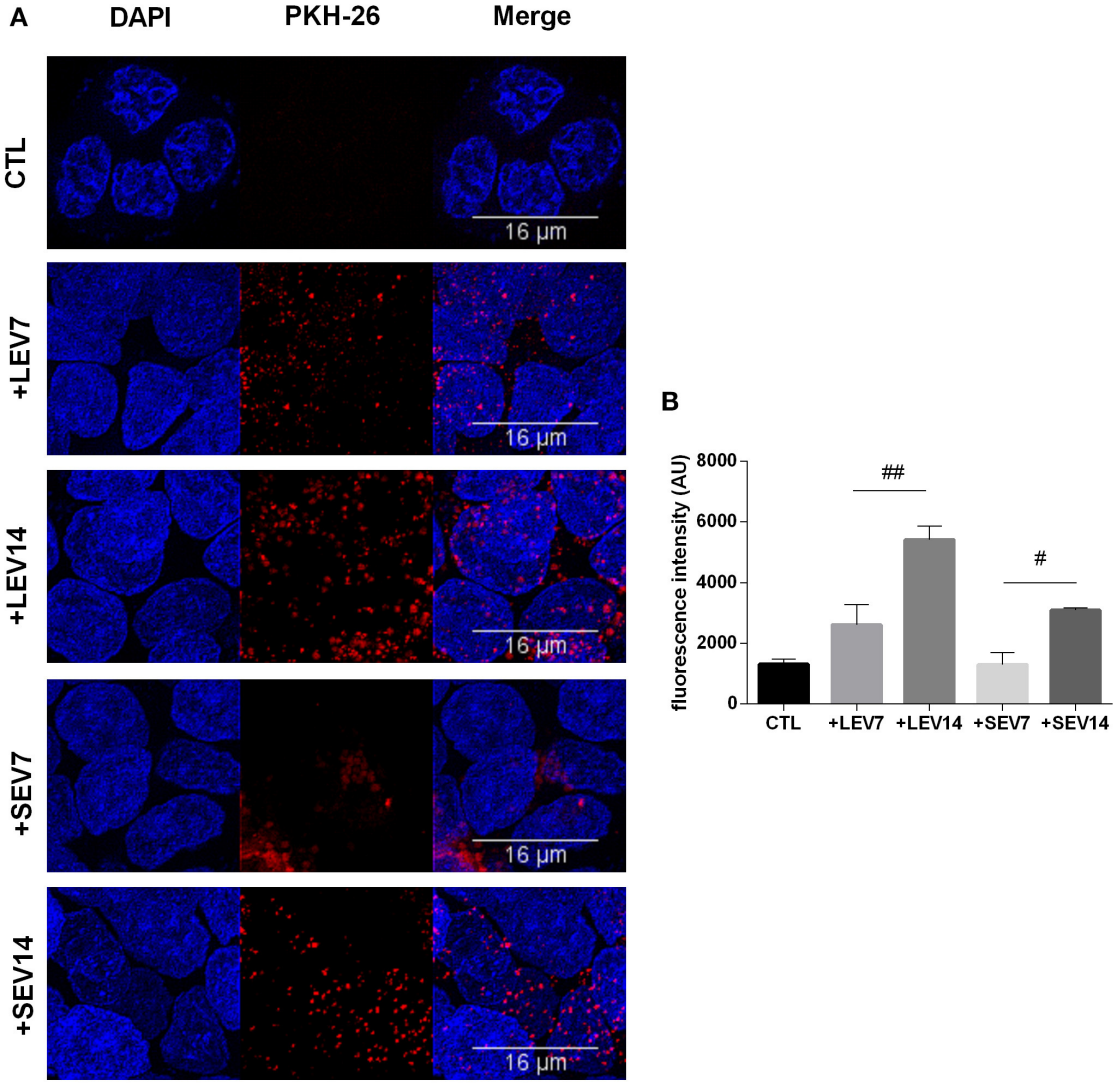
The two *G. intestinalis* EV sub-populations are efficiently taken up by Caco-2 cells. **(A)** Caco-2 cells incubated with PKH26-labeled LEVs or SEVs. **(B)** Internalized EVs were quantified by means of fluorescence intensity. Background signal was subtracted for every single image before obtaining the fluorescence intensity (Arbitrary Units); scale bars are indicated at 16 μm. Data are representative of at least three independent experiments and represented as means ± SEM. ^#^*P* = 0.05, ^*##*^*P* = 0.01; vs. the corresponding group, indicated by line.

*Giardia* derived SEVs and LEVs were analyzed for their ability to interact with host cells. Among the fluorochromes tested for EV labeling, only PKH-26 showed a homogeneous staining. Both PKH26-labeled LEVs and SEVs were incubated with Caco-2 cell monolayers for 1 h at two concentrations, 7 or 14 μg, respectively. Confocal microscopy revealed punctuated patterns of fluorescence distributed intracellularly in Caco-2 cells (Figure 7A). Both populations appeared to be taken up by the host cells in a dose-dependent manner. Despite LEV intensity internalization being observed to be higher overall, which may relate to larger vesicle size (Figure 7B), final intracellular destiny of the EVs was not determined.

## Discussion

The current study describes two distinct EV populations from *Giardia intestinalis* where large EVs (LEVs), but not small EVs (SEVs), were associated with effective parasite cell adhesion to host intestinal cells (Caco-2) (Figure 3). A role for one of the identified EV populations in host-pathogen interactions was demonstrated, as treatment *of G. intestinalis* trophozoites with pharmacological EV-inhibitors selectively decreased biogenesis of LEVs (Figure 2). EV modulatory strategies have been highlighted as an increasingly important approach in a range of pathologies (Jorfi et al., 2015; Lange et al., 2017; Catalano and O'Driscoll, 2020; Uysal-Onganer et al., 2020). Therefore, our current findings highlight that such strategies in giardiasis may be of considerable importance.

The pan-PAD-inhibitor Cl-amidine has previously been described as a potent EV-inhibitor, compared to a range of other compounds, in various cancer cells (Kosgodage et al., 2017, 2018a), as well as to affect EV-mediated microRNA export (Kosgodage et al., 2019a; Uysal-Onganer et al., 2020). Studies have also shown that PAD-inhibitors can be strategically used to sensitize cancer cells to chemotherapy (Kholia et al., 2015; Kosgodage et al., 2017) and affect cancer cell invasion (Uysal-Onganer et al., 2020). The effect of PAD-inhibitors on EV release furthermore seems to be a phylogenetically conserved pathway as PAD-inhibitors were also found to reduce EV release from bacteria and accordingly, to effectively sensitize bacteria to antibiotics (Kosgodage et al., 2019b). The EV-modulatory functions of CBD were also recently revealed, and CBD has even been found to be a more potent EV inhibitor than Cl-amidine in some cancer cell types, also to have chemosensitizing effects and showing selective inhibition on smaller or larger EVs according to cancer type (Kosgodage et al., 2018b). Furthermore, CBD was recently revealed to reduce bacterial EV release, modify proteomic content of bacterial EVs, and to sensitize certain bacteria to antibiotic treatment via this pathway (Kosgodage et al., 2019c), indicating also a phylogenetically conserved function for CBD in EV modulation. Such EV-modulatory functions, as also observed in our current study in *Giardia*, may correlate to the reported effects of cannabinoids as anti-parasitic agents, where inhibitory effects on parasite invasion and the immunosuppression of trypanosomiasis has been reported (Nok et al., 1994; Croxford et al., 2005). Cannabinoids have furthermore been shown to be effective anti-helmitics (Roulette et al., 2016), while their effects on *Giardia* have hitherto not been investigated. Our current findings may therefore be of considerable interest for putative use of CBD in giardiasis.

The field of EV research is still rapidly growing, with characterization of functions of subpopulations gaining increased attention. The complex function of LEVs revealed here in *Giardia*, suggests that their influence on phenotypes could be even more diverse than those of SEVs (Tkach et al., 2018). No biomarkers were considered in the present study, since both EV populations are enriched mixtures of vesicles that fail to contain any unique marker (Kalra et al., 2013; Vader et al., 2016) and parasite cells may have different sets of markers in their genome (Gonçalves et al., 2018; Ramirez et al., 2018).

Properties related to different functions of LEV have been studied in non-infectious models. For example, LEVs derived from cancer prostate cells contain substantially more large size dsDNA than SEVs (Vagner et al., 2018). LEVs (microvesicles) derived from platelets were also associated with polymorphonuclear leucocytes increase in adhesion (Fujimi et al., 2002). On the other hand, properties related to cellular adhesion for SEVs isolated from two cancer cell lines have also been identified while the same was not detected for LEVs (Jimenez et al., 2019). LEVs identified in *Fasciola hepatica* contained protein cargo related to digestion (cathepsin L1 zymogen), while proteomic and functional analyses identified membrane structure components and immunomodulation factors in SEVs (Cwiklinski et al., 2015).

EVs from *Giardia* have previously been studied in host-pathogen interactions. Evans-Osses et al. (2017) identified that LEVs (microvesicles) increase *in vitro* adhesion of trophozoites in Caco-2 cells, and also increase the activation of immature dendritic cells. Moyano et al. (2019) characterized a population of SEVs (exosomes), and suggested that EV release depends on ceramide and Rab11, despite parasite loss of ESCRT machinery. According to Saha et al. (2018) the parasite ESCRT is localized at the PV, the endolysosomal equivalent for *Giardia*. Wampfler et al. (2014) conducted a proteomic analysis of specified-PV and ESV content. The latter appears to assume a functional role similar to the endoplasmic reticulum, such as recruitment of ribosomes to organelle membranes (Wampfler et al., 2014). Benchimol (2004) studied the release of ESVs on giardial cell surface. They detected large granules docking in the plasma membrane. In addition, Midlej et al. (2019) investigated the release of intraluminal vesicles from trophozoites treated with CaCl_2._ Using electron microscopy techniques, they demonstrated the exocytosis of those vesicles and recovery in the supernatant. In another study, a proteomic analysis of excretory-secretory products (ESP), was conducted, including EVs of axenic cultures and cultures of trophozoite interacting with mammalian cells, identifying proteins related to metabolism, without signal peptides on EVs (Ma'ayeh et al., 2017).

Our proteomic analysis of *Giardia* EVs detected relevant virulence factors and immunogenic molecules such as cathepsin-B, arginine-metabolizing enzymes, and VSPs in both EV subpopulations. Many of the proteins found are associated with parasite cytoskeleton, such as giardins, katanin, and ankyrin repeat proteins. Due to the prominent role of proteins from cytoskeletons in parasite adhesion and virulence, we propose that *Giardia* adhesion to epithelial cells could be related to surface molecules and Disk-associated proteins, identified here to be contained in LEVs. These proteins are rich in ankyrin repeats and may contribute to attachment, protein-protein interactions, and stability (Weiland et al., 2005; Andersson et al., 2007; Nosala et al., 2018; Hagen et al., 2020). Some of the giardins detected in LEVs, such as the alpha-1 giardin, are capable of binding to glycosaminoglycans present in the intestinal epithelial monolayer, and hence can play a role in the early host–parasite interplay (Weiland et al., 2003).

*Giardia* has to survive an unfriendly environment in the small intestine, while it lacks mitochondria and a conventional ROS-scavenging enzyme, such as catalase, superoxide dismutase, and glutathione (GSH) peroxidase. Our proteome analysis also suggests that EVs carry products associated with oxidoreductase activity, such as Peroxiredoxin-1, FixW, and PFOR. These products were also identified in the work of Ansell et al. (2016). Ma'ayeh et al. (2015) investigated the transcriptome of *Giardia* isolates in oxidative stress (O_2_, H_2_O_2_). Isolate GS revealed higher levels of Peroxiredoxin-1 and other antioxidative products. In another work from the same authors (Ma'ayeh et al., 2017), a proteomic analysis of trophozoites incubated with intestinal epithelial cells detected 11 proteins with oxidoreductase activity in the WB isolate.

Our evidence of dose-dependent internalization of EVs, together with proteomic data on cytoskeleton protein enrichment, suggests that EVs may be associated with the recovery of trophozoite adhesion capacity altered with the PAD-inhibitor. Experiments with overexpression of genes in trophozoites, to be released by vesicles as blockage with monoclonal antibodies, could give an idea of whether the type of interaction is specific and assess downstream effect on molecular EV-cargo. Understanding the internalization and intracellular destiny of EVs and EV subpopulations is a future challenge for further in-depth studies.

While the majority of *Giardia* infected individuals are asymptomatic, giardiasis is a major contributor to malnutrition and growth impairment in children from developing countries (Fink and Singer, 2017). Additionally, the disease may also last for a long term chronic infection. Therefore, it is important to identify and study novel clinical strategies that can lead to host recovery. PAD-inhibitor Cl-amidine and CBD were here shown to effectively decrease parasitic EV release, which contributes to parasite adherence into intestinal epithelial cells (Caco-2 cells). They may therefore pose as novel therapeutic candidate agents for cases of chronic giardiasis.

## Conclusion

**Figure 8:**
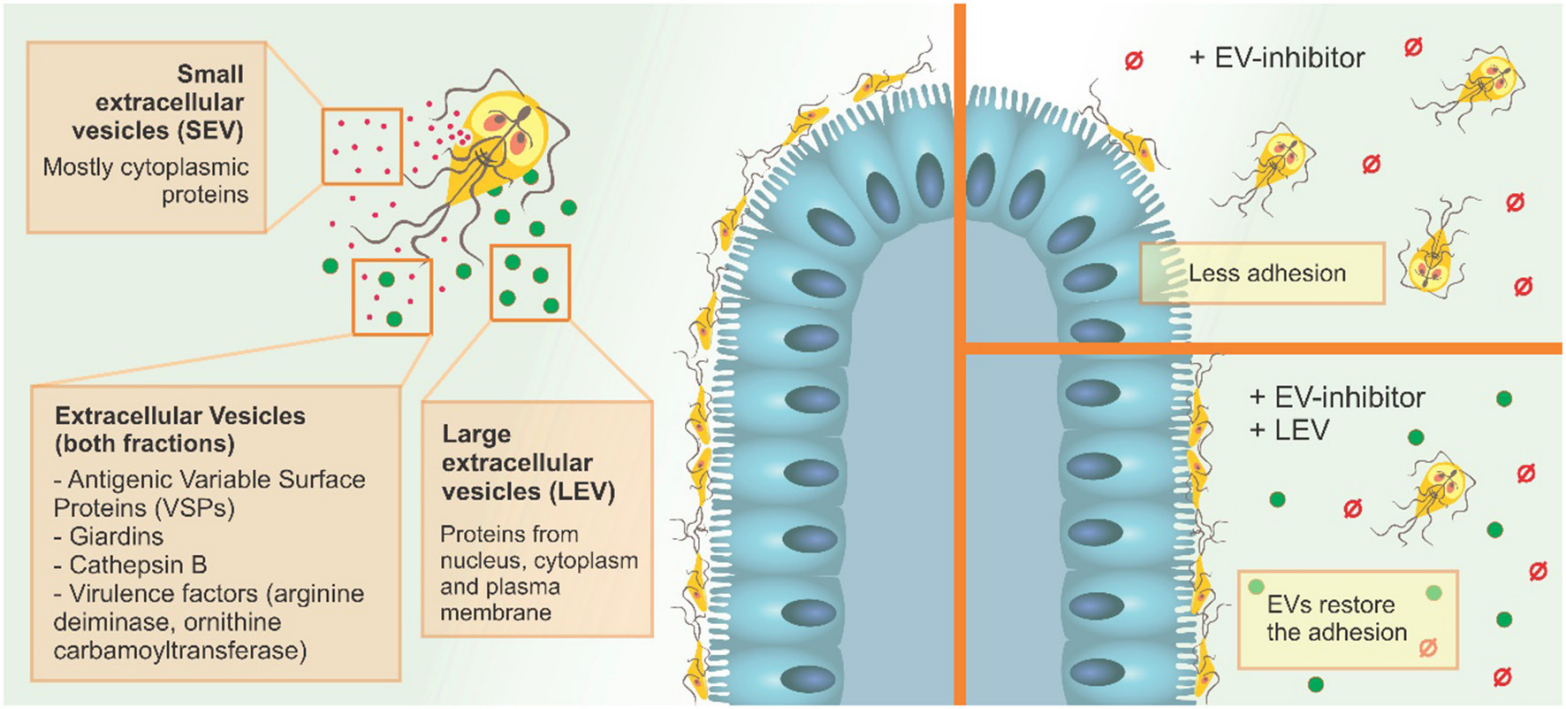
*G. intestinalis* EV sub-populations have different protein content, and differentially restore adhesion of trophozoites following EV-inhibitor treatment.

Our results suggest that the two EV populations identified in *G. intestinalis* so far, LEVs and SEVs, have distinct protein content and functions in the phenotype of this pathogen and can be selectively modulated using PAD-inhibitors and CBD (Figure 8). Since adhesion in the epithelial intestine is fundamental to parasite fitness and invasion, and LEVs clearly aid this process, the use of targeted EV–inhibitors, such as Cl-amidine identified here, can be used to selectively interfere with EV secretion, allowing novel treatment strategies in the control of giardiasis.

## Data Availability Statement

The original contributions presented in the study are publicly available. This data can be found here: https://www.ncbi.nlm.nih.gov/ Project accession: PXD018460.

## Author Contributions

BG, BS, IVR, JS, and VF made experiments. BG, SL, GP, and MIR wrote manuscripts. All authors planned experiments.

## Conflict of Interest

The authors declare that the research was conducted in the absence of any commercial or financial relationships that could be construed as a potential conflict of interest.

## Supplementary Material

Supplementary Figure 1 EV protein estimation from parasite and mammalian cells (Caco-2). Data are representative of at least three independent experiments and represented as means ± SEM. ^#^*P* = 0.05; vs. the corresponding group, indicated by line.

Supplementary Figure 2 Host-pathogen assay after treatment with PAD-inhibitors and EVs subjected to heat inactivation. Data are representative of at least three independent experiments and represented as means ± SEM. ^#^*P* = 0.05; vs. the corresponding group, indicated by line. ^***^*P* = 0.001; compared to control group (CTL).

Supplementary Figure 3 Growth curve for culture confluence of trophozoite after exposure to 100 μM Cl-am or 10 μM CBD, following 96 h incubation. Data are representative of at least three independent experiments and represented as means ± SEM. ns, not significant.

Supplementary Figure 4 Host-pathogen assay treated with a protease inhibitor and LEVs. Data are representative of at least three independent experiments and represented as means ± SEM. ^#^*P* = 0.05; vs. the corresponding group, indicated by line. ^**^*P* = 0.01; compared to control group (CTL).

Supplementary Table 1 Proteomic content in SEVs and LEVs.

Supplementary Table 2 Products associated with cytoskeleton (microtubules), ventral disk, median body and flagella, and oxidative stress responses found in LEVs.
