# Supplementary figures and images for: Peptidylarginine Deiminase Inhibition Abolishes the Production of Large Extracellular Vesicles From Giardia intestinalis, Affecting Host-Pathogen Interactions by Hindering Adhesion to Host Cells
Source: Front Cell Infect Microbiol. 2020 Sep 23;10:417. doi: 10.3389/fcimb.2020.00417 (PMC7539837; doi:10.3389/fcimb.2020.00417)

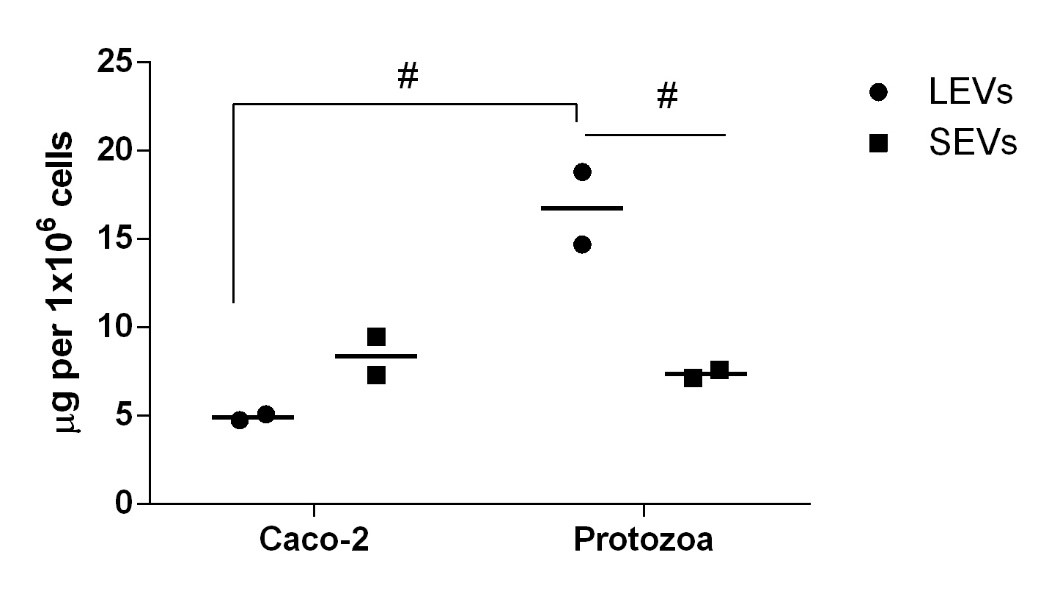

Supplement: Supplementary Figure 1 — EV protein estimation from parasite and mammalian cells (Caco-2). Data are representative of at least three independent experiments and represented as means ± SEM. #P = 0.05; vs. the corresponding group, indicated by line. [file Image_1.jpg]

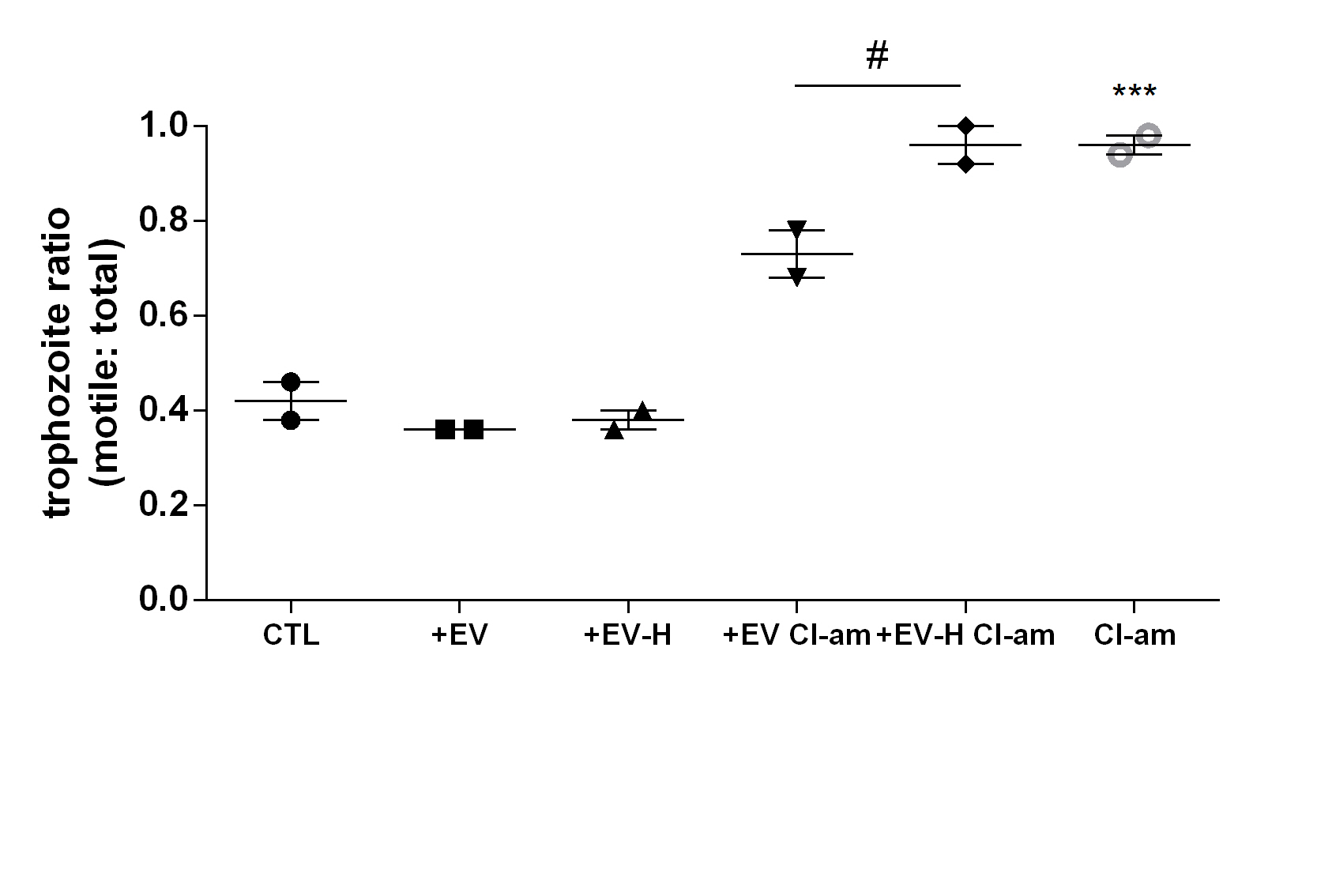

Supplement: Supplementary Figure 2 — Host-pathogen assay after treatment with PAD-inhibitors and EVs subjected to heat inactivation. Data are representative of at least three independent experiments and represented as means ± SEM. #P = 0.05; vs. the corresponding group, indicated by line. ***P = 0.001; compared to control group (CTL). [file Image_2.jpg]

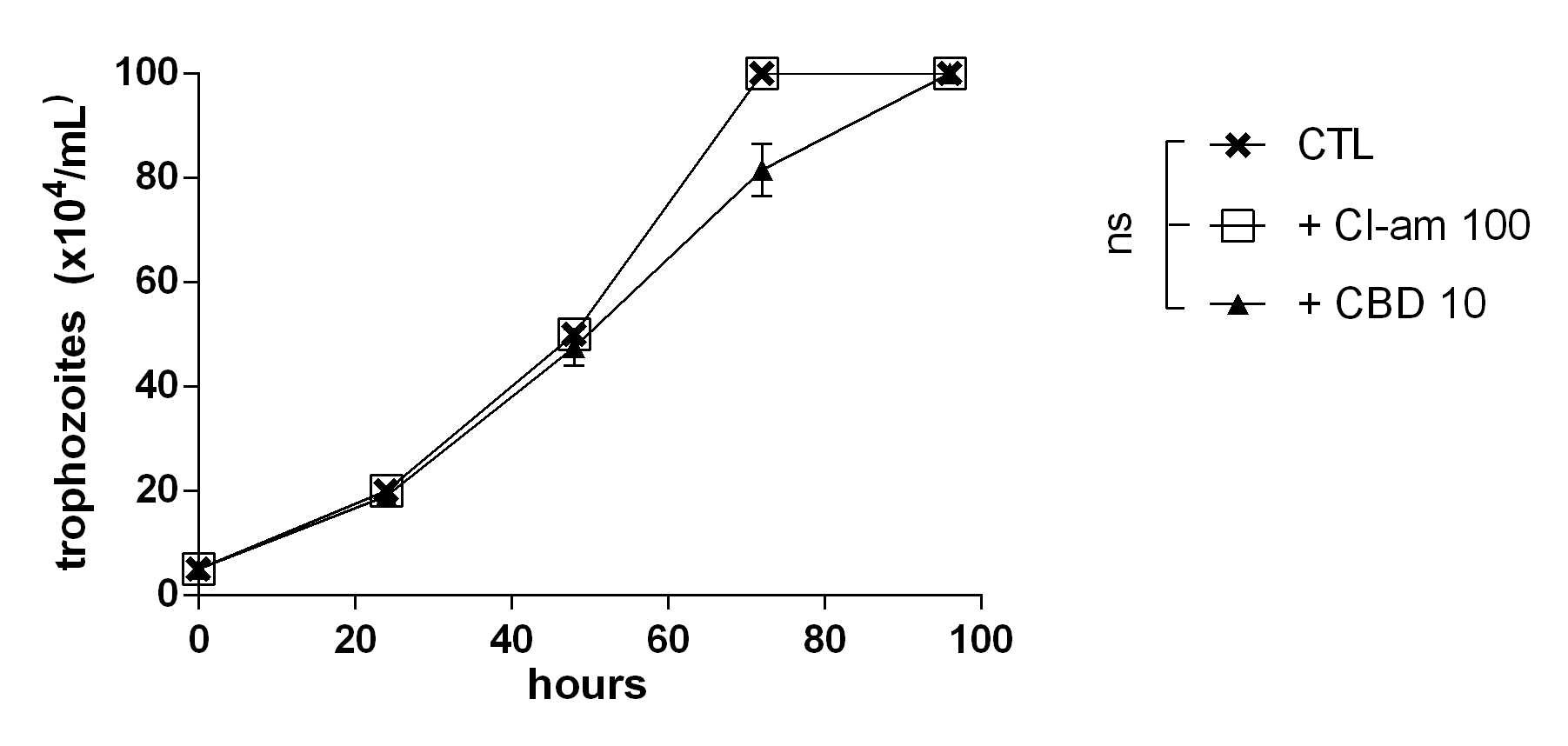

Supplement: Supplementary Figure 3 — Growth curve for culture confluence of trophozoite after exposure to 100 μM Cl-am or 10 μM CBD, following 96 h incubation. Data are representative of at least three independent experiments and represented as means ± SEM. ns, not significant. [file Image_3.jpg]

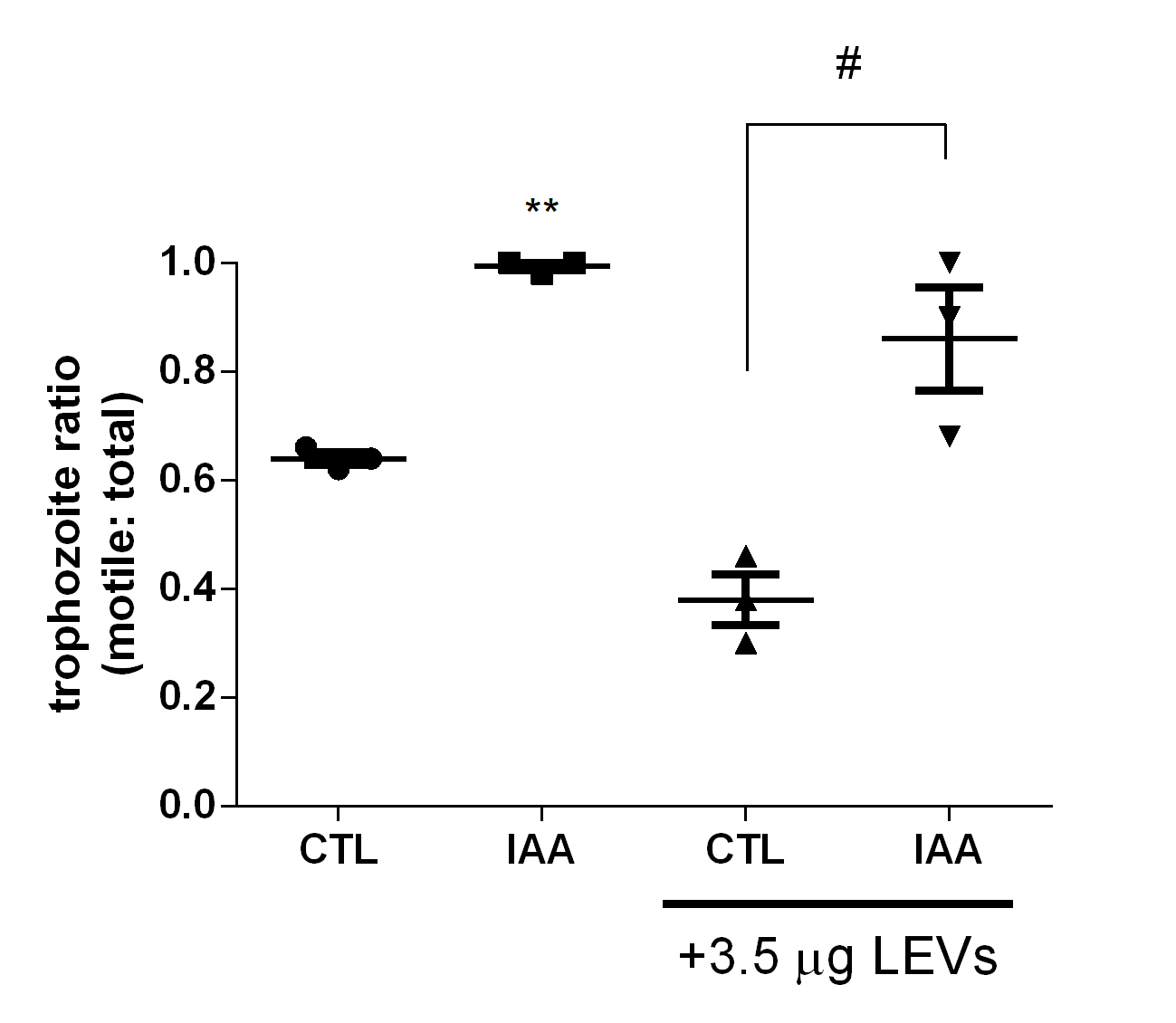

Supplement: Supplementary Figure 4 — Host-pathogen assay treated with a protease inhibitor and LEVs. Data are representative of at least three independent experiments and represented as means ± SEM. #P = 0.05; vs. the corresponding group, indicated by line. **P = 0.01; compared to control group (CTL). [file Image_4.jpg]
